# Study on Water-Soluble Phenolic Resin Gels for High-Temperature and High-Salinity Oil Reservoir

**DOI:** 10.3390/gels9060489

**Published:** 2023-06-14

**Authors:** Yunling Ran, Guicai Zhang, Ping Jiang, Haihua Pei

**Affiliations:** School of Petroleum Engineering, China University of Petroleum (East China), Qingdao 266580, China; upcrhelm20@163.com (Y.R.); jiangping@upc.edu.cn (P.J.); peihaihua@upc.edu.cn (H.P.)

**Keywords:** low-cost gel, AM-AMPS copolymer, high-temperature and high-salinity resistant gel, oil displacement experiment

## Abstract

High water cut of produced fluid is one of the most common problems in reservoir development. At present, injecting plugging agents and other profile control and water plugging technologies are the most widely used solutions. With the development of deep oil and gas resources, high-temperature and high-salinity (HTHS) reservoirs are becoming increasingly common. Conventional polymers are prone to hydrolysis and thermal degradation under HTHS conditions, making polymer flooding or polymer-based gels less effective. Phenol–aldehyde crosslinking agent gels can be applied to different reservoirs with a wide range of salinity, but there exist the disadvantage of high cost of gelants. The cost of water-soluble phenolic resin gels is low. Based on the research of former scientists, copolymers consisting of acrylamide (AM) and 2-Acrylamido-2-Methylpropanesulfonic acid (AMPS) and modified water-soluble phenolic resin were used to prepare gels in the paper. The experimental results show that the gelant with 1.0 wt% AM-AMPS copolymer (AMPS content is 47%), 1.0 wt% modified water-soluble phenolic resin and 0.4 wt% thiourea has gelation time of 7.5 h, storage modulus of 18 Pa and no syneresis after aging for 90 days at 105 °C in simulated Tahe water of 22 × 10^4^ mg/L salinity. By comprehensively comparing the effectiveness of the gels prepared by a kind of phenolic aldehyde composite crosslinking agent and modified water-soluble phenolic resin, it is found that the gel constructed by the modified water-soluble phenolic resin not only reduces costs, but also has shorter gelation time and higher gel strength. The oil displacement experiment with a visual glass plate model proves that the forming gel has good plugging ability and thus improves the sweep efficiency. The research expands the application range of water-soluble phenolic resin gels, which has an important implication for profile control and water plugging in the HTHS reservoirs.

## 1. Introduction

The problem of high water cut in oilfield development needs to be solved urgently, as it has the potential to reduce the utilization efficiency of injected water. A large amount of injected water flows through the high permeability layer, resulting in premature water emergence in oilfield development and a low sweep efficiency of the injected water [1]. To solve the above problems, common method is to inject selective plugging agents into high-permeability channels such as fractures [2,3]. Phenolic gel is one of the most commonly used plugging agents, the use of which is mainly divided into two methods: one is to make gelants by adding phenol and aldehyde solutions to the polymer solutions [4,5,6,7,8,9,10,11,12,13,14]; the other is to directly add a water-soluble phenolic resin solution into the polymer solutions [2,3,15,16,17,18,19,20,21,22,23]. 

The first type of gels has been widely studied and applied in reservoirs with different temperatures and salinities [4,5,6,7,8,9,10,11,12,13,14]. This paper focuses on the second kind of gels. Compared with the phenol–aldehyde crosslinking system gels, the latter can be prepared at a low cost. At present, some scientists have studied some gelling behaviors using water-soluble phenolic resin to replace the phenol–aldehyde crosslinking agent system [15,16,17,18,19,20,21,22,23]. According to the microstructure of chrome gel and phenolic resin gel prepared by Zhang et al. [16], the network structure of the phenolic resin gel is more prone to deformation under external forces. At high magnification rates, the phenolic gel network is more developed than the dendritic cluster structure of the chrome gel. Under the conditions of low temperature and salinity, the properties of the gels formed by cross-linking water-soluble phenolic resin with different polymers were also studied. Shang et al. [17] prepared a gel system formed by partially hydrolyzed polyacrylamide (HPAM) crosslinked with organic chromium and water-soluble phenolic resin at a temperature lower than 80 °C, a salt concentration lower than 16,000 mg/L, and a pH value between 6 and 8. The blocking rate of the system in the simulated core was reported to be 96%. Cui et al. [18] determined that gels with good thermal stability and shear resistance could be obtained when the concentration of HPAM was greater than 600 mg/L and the concentration of water-soluble phenolic resin was more than 1400 mg/L. Zhao et al. [19] selected phenolic resin cross-linked non-ionic PAM to prepare gels for deep profile control. The salinity in their experiment was 492.08 mg/L, and the experiment temperature was 80 °C. Gu et al. [20] used phenolic resin to crosslink non-ionic polyacrylamide to prepare gels and observed that when the concentration of the polymer and the crosslinking agent increased, so did the gels’ viscosity and strength, but the viscosity and strength decreased when the concentration of salt or pH value increased. The phenolic resin gel system could maintain good stability at 95 °C under alkaline conditions with a salt concentration lower than 30,000 mg/L or a pH value greater than 7.

In the past five years, water-soluble phenolic resin has been further applied in oil fields. In 2019, Ge et al. [2] and Wu et al. [3] prepared a water-soluble phenolic resin gel at 70 °C for the low porosity and ultra-low permeability fractured reservoir in the Honghe Oilfield. The gel had good long-term stability after 180 days of heat treatment, and the strength was more than 10 Pa. In 2022, the impact of shear rate on the gelling behavior of phenolic resin gel in porous media was investigated by Yu et al. [21]. The findings revealed that the dynamic gel time was minimally influenced by the injection speed, whereas the gel strength was significantly affected. Guo et al. [22] used partially hydrolyzed polyacrylamide (HPAM) as the main agent, water-soluble phenolic resin (WSPR) as the crosslinking agent, and nano-SiO_2_ as the stabilizer. The gelation time could be varied between 3 h and 23 h at 110 °C and 12,124 mg/L of salinity, and within 180 days, the gel’s stability was still excellent. Xu et al. [23,24] chose AM-AMPS copolymer as the gel-forming agent and studied the plugging performance of a water-soluble phenolic resin gel system under the conditions of a salt concentration of 41,110 mg/L and a temperature range of 80–90 °C. Qu et al. [25] presented a gel with gelation time of 26–34 h at 55 °C using polyacrylamide, chromium acetate and water-soluble phenolic resin for the purpose of controlling water coning. In 2023, Zhi et al. [26] prepared a weak gel that demonstrated excellent resistance to temperature and salt by utilizing a crosslinking agent in conjunction with the phenolic resin when the salinity was 40,300.86 mg/L at 120 °C. From the above studies, it can be found that during the preparation of gels, most of the water-soluble phenolic resins are used at temperatures between 70 °C and 90 °C, and the reservoir salinity is usually lower than 4 × 10^4^ mg/L. It is because water-soluble phenolic resins would precipitate and stick to walls in high salinity water, resulting in poor stability and unsatisfactory gelling properties. 

In view of this, copolymers consisting of acrylamide (AM) and 2-acrylamido-2-methylpropanesulfonic acid (AMPS) and a modified water-soluble phenolic resin are used to prepare gels in this paper. The experimental results show that the gelant with 1.0 wt% AM-AMPS copolymer (AMPS content is 47%), 1.0 wt% modified water-soluble phenolic resin, and 0.4 wt% thiourea has a gelation time of 7.5 h, a storage modulus of 18 Pa, and no syneresis after aging for 90 days at 105 °C in simulated Tahe water with a salinity of 22 × 10^4^ mg/L. That is to say, compared to the application conditions of the gels prepared by other scientists using water-soluble phenolic resin, this gel can be used in a higher salinity condition. The state changes of the modified water-soluble phenolic resin gel added to simulated Tahe water were observed, and the plugging performance of the modified water-soluble phenolic resin gel in porous media was evaluated in the experiment which proved that the forming gel had good stability and plugging ability. This research expands the application range of water-soluble phenolic resin gels, which has an important implication for profile control and water plugging in the HTHS reservoirs.

## 2. Results and Discussion

### 2.1. Evaluation of Gelation Performances of the Modified Soluble Phenolic Resin Gels

Phenol–aldehyde crosslinking agent gels can be applied to different reservoirs with a wide range of salinities, but they have the disadvantage of high cost of gelants. The cost of water-soluble phenolic resin is relatively low, so we are trying to use it to prepare a cheaper gel to meet the needs of the HTHS reservoirs. 

The water-soluble phenolic resin is prone to self-polycondensation at room temperature and has poor solubility under high salinity. Clearly, this is not a good situation for making high-temperature and high-salinity resistant gels. Liu et al. [27] prepared a modified water-soluble phenolic resin by adding p-hydroxybenzoic acid. Their research shows that when the dosage of p-hydroxybenzoic acid is 10%, the phenolic resin can stably exist in Tahe water for more than 4 weeks at 50 °C. In other words, the modified water-soluble phenolic resin has excellent solubility in high-salinity water. Additionally, the research papers by Zhang et al. [28] and Guo et al. [29] showed that AM-AMPS copolymer could enhance the long-term stability of the prepared gel and reduce its syneresis rate in the HTHS environment.

Referring to the preparation method of conventional water-soluble phenolic resin, adding AM-AMPS copolymer solutions to the water-soluble phenolic resin modified by adding p-hydroxybenzoic acid may generate a uniformly high-strength gel to achieve a plugging effect. AM-AMPS copolymers with AMPS content of 0%, 25%, and 47% are referred to as PAM, AM-AMPS 30 and AM-AMPS 50, respectively. They were selected as gelling agents. The modified water-soluble phenolic resin was used as a crosslinking agent, and thiourea with a mass fraction of 0.4 wt% was added as a stabilizer to investigate the gelling performance of the prepared gels in simultated Tahe water at a temperature of 105 °C.

#### 2.1.1. Gelation Time of the Modified Soluble Phenolic Resin Gels

**Figure 1:**
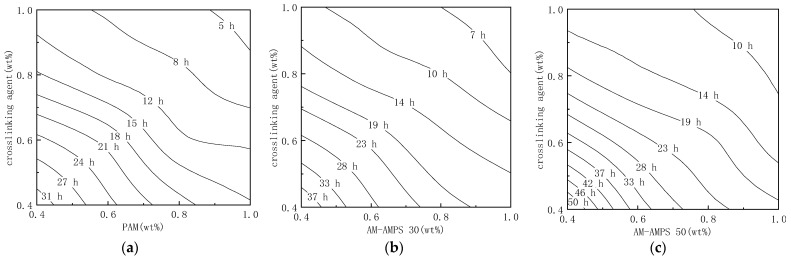
Gel formation time of different component gels aged at 105 °C: (**a**) Gel formation time of PAM and modified soluble phenolic resin; (**b**) Gel formation time of AM-AMPS 30 and modified soluble phenolic resin; (**c**) Gel formation time of AM-AMPS 50 and modified soluble phenolic resin.

At a temperature of 105 °C and a salinity of 22 × 10^4^ mg/L, copolymers with different AMPS content were crosslinked with the modified water-soluble phenolic resin. The gelation time of the prepared gels is shown in Figure 1.

It is evident that the gelation time and the mass fraction of the polymer and crosslinking agent have a negative relationship when the content of AMPS is constant. When the mass fractions of the polymer and crosslinking agent are both 0.4–1.0 wt%, the gelation time with PAM as the main agent is 4–32 h; the gelation time of an AM-AMPS copolymer with AMPS content of 25% as the main agent is 5–40 h; and the gelation time of an AM-AMPS copolymer with AMPS content of 47% as the main agent is 7.5–55 h. That is to say, when the AMPS content increases, the gelation time becomes significantly prolonged, which is similar to the results of Xu et al. [23,24].

#### 2.1.2. Storage Moduli of the Modified Soluble Phenolic Resin Gels

**Figure 2:**
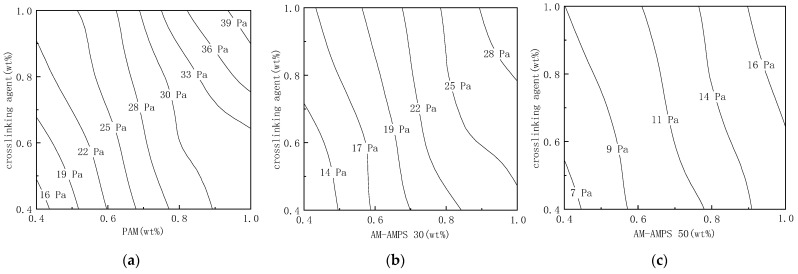
Storage modulus of different component gels aged at 105 °C: (**a**) Storage modulus of PAM and modified soluble phenolic resin; (**b**) Storage modulus of AM-AMPS 30 and modified soluble phenolic resin; (**c**) Storage modulus of AM-AMPS 50 and modified soluble phenolic resin.

As seen in Figure 2, the storage moduli of the gels prepared by crosslinking polymers with different AMPS content and the modified water-soluble phenolic resin at 105 °C after 5 days were measured. It can be seen that the strength has a good positive association with the mass fraction of the polymer and crosslinking agent. When the mass fractions of the polymer and crosslinking agent are both 0.4–1.0 wt%, the strength of AM-AMPS gels composed of AMPS monomer content of 0%, 25% and 47%, respectively, is 14–40 Pa, 10–30 Pa, and 5–18 Pa. That is to say, as the AMPS content in the polymer increases, the gel strength weakens, which is similar to the research results of Wang et al. [15]. 

#### 2.1.3. Long-Term Stability of the Modified Soluble Phenolic Resin Gels

**Figure 3:**
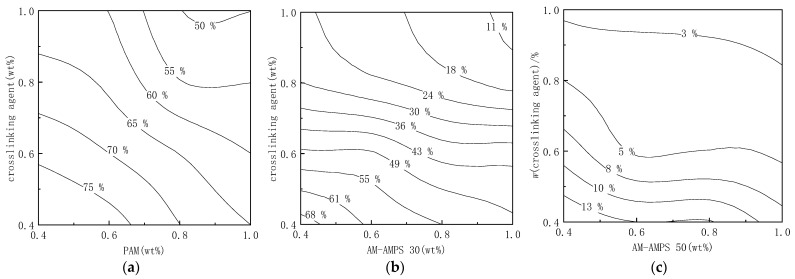
Syneresis rate of different component gels aged at 105 °C: (**a**) Syneresis rate of PAM and modified soluble phenolic resin; (**b**) Syneresis rate of AM-AMPS 30 and modified soluble phenolic resin; (**c**) Syneresis rate of AM-AMPS 50 and modified soluble phenolic resin.

The stability of the gels was characterized by the syneresis rate at 105 °C after 90 days. The syneresis rate of the gels prepared by crosslinking copolymers of different AMPS content with the modified water-soluble phenolic resin is shown in Figure 3.

As can be observed, the stability of gels with different AMPS content is positively correlated with the mass fraction of copolymer and crosslinking agents. As the mass fraction of copolymer and crosslinking agents increases, the syneresis rate of gels decreases. When the mass fractions of the polymer and crosslinking agent are both 0.4–1.0 wt%, the syneresis rate of the gels with PAM polymer as the main agent aged at 105 °C for 90 days is between 50% and 80% (exceeding 50%). The syneresis rate of the gels mainly composed of an AM-AMPS copolymer with AMPS content of 25% varies from 10% to 70%. The gel with a high mass fraction of copolymer and crosslinking agent has good stability and a low syneresis rate. The syneresis rate of the gel prepared with 1.0 wt% copolymer and 1.0 wt% crosslinking agent is only 10%, and the syneresis rate of the gel prepared with a 0.4 wt% copolymer and a 0.4 wt% crosslinking agent is 70%. In addition, the increase in AMPS content also enhances the stability of the gel. The syneresis rate of the gels using a copolymer with AMPS content of 47% as the main agent is 0–15%, indicating a significant improvement in the stability of the gel.

In summary, examining the gelation performance of the gels prepared by the modified water-soluble phenolic resin and AM-AMPS copolymer, it becomes clear that with the increase in the AMPS content, the gelation time of the gels becomes longer and the long-term stability increases, but the strength of the gel decreases. This is related to the structure of the copolymer. The crosslinking mechanism of the gel is the polycondensation of phenolic resin and amide group in polymer chains [1]. As the copolymer’s AMPS content rises, the amide group content correspondingly reduces, resulting in a longer crosslinking time and weaker gel strength. However, the rate of hydrolysis and degradation of the copolymer at high temperature slows down. Therefore, the long-term stability of the prepared gel is enhanced.

**Figure 4:**
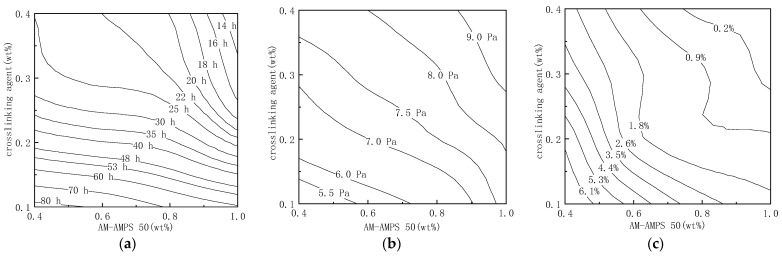
Performance of different component gels aged at 105 °C: (**a**) Gel formation time of AM-AMPS 50 and phenolic aldehyde composite crosslinking agent; (**b**) Storage modulus of AM-AMPS 50 and phenolic aldehyde composite crosslinking agent; (**c**) Syneresis rate of AM-AMPS 50 and phenolic aldehyde composite crosslinking agent.

The performance of the prepared gel using the AM-AMPS copolymer and the phenolic aldehyde composite crosslinking agent was evaluated at 105 °C and a salinity of 22 × 10^4^ mg/L, as shown in the following Figure 4. The copolymer with AMPS monomer ratio of 47% was selected as the gelling agent, the phenolic crosslinking agent was hydroquinone, and the aldehyde crosslinking agent was urotropine.

It can be seen that the gelation time is 12–83 h, the strength after 5 days is 4.9–9.8 Pa, and the dehydration rate after 90 days is only 0–7%. When the mass fraction of AM-AMPS copolymer is 1.0 wt%, the hydroquinone is 0.4 wt%, and the urotropine is 0.8 wt%, the gelation time of the prepared phenolic aldehyde crosslinking agent gels can be shortened to 12 h, and the storage modulus after 5 days of aging is 9.8 Pa (Sydansk’s gel strength code of “G”: when the ampoule is inverted, the gel flows down to about half of the ampoule). The gel has good stability and does not dehydrate within 90 days, making it suitable for the harsh conditions of high temperature and high salinity in Tahe Oilfield. 

Using a mass fraction of 1.0 wt% AM-AMPS copolymer (AMPS content is 47%), 1.0 wt% modified water-soluble phenolic resin, and 0.4 wt% thiourea as the formula, a low-cost gel with gelation time of 7.5 h, storage modulus of 18 Pa, and no syneresis at 105 °C after 90 days can be prepared. By comprehensively comparing the performance of the gel prepared by a phenolic aldehyde composite crosslinking agent and the modified water-soluble phenolic resin, it can be found that the gel constructed by the modified water-soluble phenolic resin not only reduces costs, but also has a shorter gelation time and higher gel strength, albeit with slightly poorer stability. 

This may happen because the crosslinking reaction between the water-soluble phenolic resin and the polymer skips the early process of the phenol–aldehyde reaction, leading to a shortened crosslinking reaction time. In addition, the water-soluble phenolic resin has a larger conformation and a certain degree of rigidity, resulting in the formation of gels with higher strength.

### 2.2. Evaluation of Plugging Performance of Modified Water-Soluble Phenolic Resin Gel

#### 2.2.1. Gel Strength after Adding Water

**Figure 5:**
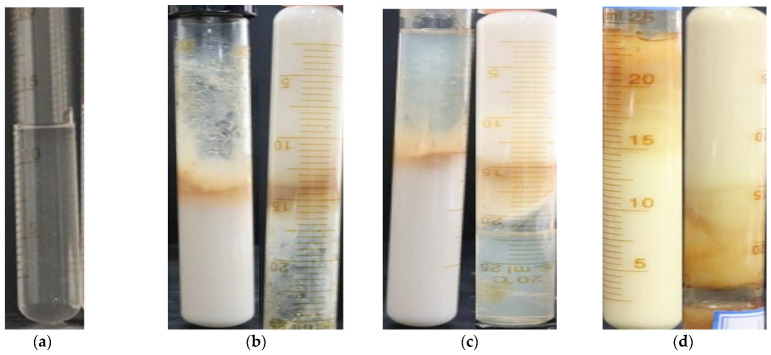
State changes of the modified water-soluble phenolic resin gel after adding Tahe water: (**a**) the state of the gel for 0 h; (**b**) the state of the gel for 24 h; (**c**) the state of the gel for 7 days; (**d**) the state of the gel for 30 days.

After the modified water-soluble phenolic resin gel is injected into the formation, it first comes into contact with a large amount of formation water. In order to investigate the effect of high-salinity Tahe water on the performance of the gel under 105 °C conditions, simulated formation water was added to the gel in a 1:1 volume ratio to observe the strength changes in the gel. The results of the experiment can be seen in Figure 5.

From the results, it can be seen that for the modified water-soluble phenolic resin gel, after the Tahe simulated formation water was added, the gel strength level was H (when the ampoule is inverted, only the surface of the gel is slightly deformed) after 30 days, and it could still maintain good stability.

#### 2.2.2. Plugging Ability of Gel in Porous Media

**Figure 6:**
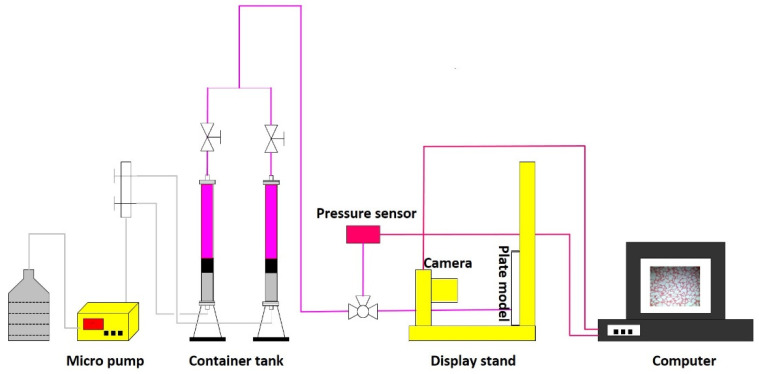
Visualized plate model displacement experiment.

The performance of the most stable water-soluble phenolic resin gel (1.0 wt% AM-AMPS 50 + 1.0 wt% modified water-soluble phenolic resin + 0.4 wt% thiourea) was evaluated by conducting a displacement experiment in a self-made visual glass flat plate model filled with sand. The experimental process, which is shown in Figure 6, can be divided into three steps: first, after the model is saturated with oil, primary water flooding is carried out until the water cut reaches 94%; then, 0.3 PV of the modified water-soluble phenolic resin gelant is injected and allowed to sit for 24 h; finally, the subsequent water drive is conducted until the water cut reaches 98%.

**Figure 7:**
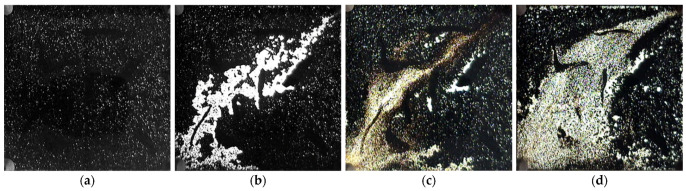
Residual oil distributions at different stages of heterogeneous model: (**a**) Residual oil distribution before first water drive; (**b**) Residual oil distributions after first water drive; (**c**) Residual oil distributions after gel formation; (**d**) Residual oil distributions after subsequent water drive.

The distributions of residual oil at different stages were observed using a heterogeneous visual sand-filling model, as shown in Figure 7. From Figure 7b, it can be seen that during water injection development under heterogeneous reservoir conditions, water gives priority to flow in the high permeability zones to form fluid channeling, and the color becomes significantly lighter because of the decrease in oil content after the primary water drive. The analysis of Figure 7d reveals that the gelling agent successfully forms a robust gel in the central region of the reservoir, thereby inducing a plugging effect. The gel formation obstructs the “dominant channel” in the high permeability zones, leading to the displacement of oil by water in the low-permeability section. Consequently, the oil sand in the low permeability zones exhibits a lighter color, indicating an enhancement in the sweep efficiency and an improvement in the recovery of crude oil due to the injection of gel.

**Figure 8:**
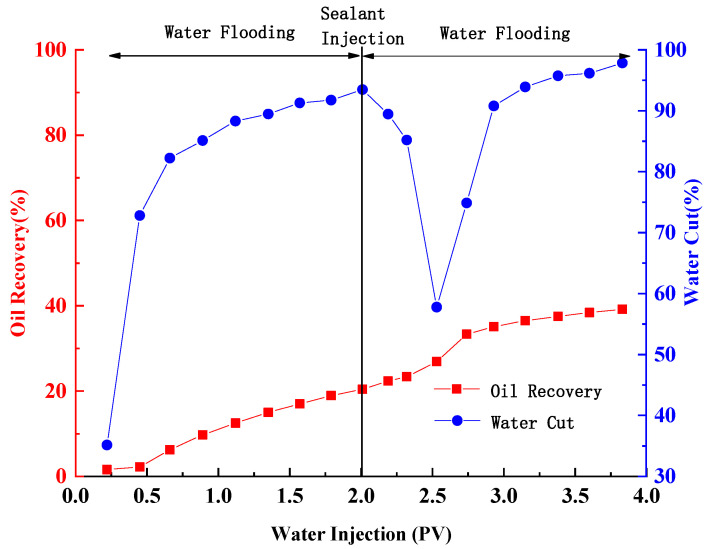
Production curve of heterogeneous model.

Figure 8 illustrates the correlations of oil recovery and water cut with injected volume of water in the experiment. The results indicate that an increase in water injection leads to a gradual rise in oil recovery. At water cut of 93.48%, the cumulative oil production amounted to 16.3 mL, resulting in a recovery rate of 20.45%. After the gel is injected, it occupies the pores in the highly permeable zone of the model. Subsequently, during the secondary water flooding phase, water infiltrates the low permeability layer, displacing some of the oil before creating a new water flooding path. The displacement of oil by water flooding results in a gradual improvement in recovery. After water cut of 97.84% is reached, the cumulative oil production amounts to 31.22 mL, and the recovery rate reaches 39.17%. The introduction of gel during secondary water flooding leads to an increase in oil recovery by 18.72%.

## 3. Conclusions

Different gels were prepared using the modified water-soluble phenolic resin and AM-AMPS copolymers with AMPS content of 0%, 25%, and 47%, respectively. The experimental results indicate that the gelation time decreases with the increase in the mass fraction of the copolymer and crosslinking agent when the content of AMPS is constant. Furthermore, with the increase in the AMPS content of the copolymer, the gelation time is significantly prolonged, the strength of the gel decreases, but Its long-term stability improves. 

The optimal formulation for the gelling solution is 1.0 wt% AM-AMPS copolymer (AMPS content is 47%), 1.0 wt% modified water-soluble phenolic resin, and 0.4 wt% thiourea. The high-performance gel with a gelation time of 7.5 h, a storage modulus of 18 Pa, and no syneresis after 90 days can be prepared in the simulated Tahe water at 105 °C and a salinity of 22 × 10^4^ mg/L. By comprehensively comparing the performance of the gel prepared by a phenolic aldehyde composite crosslinking agent and the modified water-soluble phenolic resin, it can be found that the gel constructed by the modified water-soluble phenolic resin not only reduces costs, but also has a shorter gelation time and higher gel strength, though it has slightly poorer stability. 

The displacement experiment with a visual glass plate model has proved that the formed gel has good plugging ability and can improve the sweep efficiency. This research expands the application range of water-soluble phenolic resin gels, which has an important implication for profile control and water plugging in high-temperature and high-salinity reservoirs.

## 4. Materials and Methods

### 4.1. Materials

A series of analytical pure chemical reagents such as NaCl, CaCl_2_, MgCl_2_, NaHCO_3_, formaldehyde, phenol, sodium hydroxide, and p-hydroxybenzoic acid were purchased from Sinopharm Chemical Reagent Co., Ltd. (Shanghai, China); hydroquinone, thiourea, and urotropine were provided by Aladdin Biochemical Technology Co., Ltd. (Shanghai, China); PAM was provided by Anhui Jucheng Fine Chemical Co., Ltd. (Huaibei, China); AM-AMPS copolymers (AMPS content is 25% and 47%, respectively) were prepared by an aqueous solution polymerization method, with the initiator being a redox system consisting of ammonium persulfate and sodium bisulfite; nitrogen was provided by Qingdao Xinkeyuan Technology Co., Ltd. (Qingdao, China)

The main instruments are shown in Table 1.

### 4.2. Methods

#### 4.2.1. Preparation of the AM-AMPS Copolymer Solution

The molecular weight of a water-soluble AM-AMPS copolymer is relatively large, and its dissolution in water requires a certain amount of time. The AM-AMPS copolymer with AMPS content of 47% was taken as an example, and the AM-AMPS copolymer was added to the simulated Tahe water to prepare a certain mass fraction of the copolymer solution, which was stirred slowly for 24 h, left to swell for 72 h, and then stirred for an additional 2 h.

#### 4.2.2. Synthesis Method of the Modified Water-Soluble Phenolic Resin

At present, many scientists have synthesized soluble phenolic resins using phenol and formaldehyde in the laboratory. The two-step catalytic method is a commonly used method with similar steps [30,31,32]. The thermostatic water bath is controlled at 50 °C, with the molar ratio of formaldehyde to phenol set at 3:1, and 8.0% NaOH of the total mass of formaldehyde and phenol added as the catalyst. Then, the formaldehyde is slowly dropped into the chemical reactor using a separatory funnel, with the dropping time controlled at 30 min. The temperature is quickly raised to 70 °C after the synthesis of water-soluble phenolic resin, 10.0% p-hydroxybenzoic acid of the total mass of formaldehyde and phenol is added to the water-soluble phenolic resin and the mixture is stirred thoroughly for 40 min. Finally, the modified water-soluble phenolic resin with good transparency is obtained.

#### 4.2.3. Preparation Method of Water-Soluble Phenolic Resin Gel

The gel consists of a copolymer solution, the crosslinking agent, or a certain stabilizer. Firstly, at room temperature, the prepared water-soluble phenolic resin is added to the simulated Tahe water according to the designed gel formula, and a mass fraction of 0.4 wt% thiourea is added as a stabilizer. Then, AM-AMPS copolymer is added while stirring, and a uniform gel solution is obtained after stirring. A total of 20 g of the gelling solution is weighed and injected into an ampoule, sealed with an alcohol blowtorch, and then placed in a thermostatic oven at 105 °C for heat treatment to form the gel.

#### 4.2.4. Determination of Gelation Time and Strength of Gel

The gelation time and gel strength were qualitatively determined using the bottle test method, following Sydansk’s gel code [33]. The gelation time was defined as the duration required for the gels to attain Sydansk’s code of “D” (when the ampoule is inverted, only a small part of the gel is difficult to flow to the bottom). After sealing the sample, the gelation time and gel strength were observed at ambient temperature. In addition, the storage modulus of the gel was evaluated using a rheometer at a temperature of 25 °C. The setting method of the instrument can be referred to our previous article [34].

#### 4.2.5. Method for Determining the Syneresis Rate of Gel

The evaluation of the long-term stability of a gel is mainly based on the syneresis rate of a gel as a reference indicator; when the syneresis rate of the gel is high after aging under the same temperature conditions, it can be considered that the long-term stability of the gel is poor, whereas if the syneresis rate is low or there is no syneresis phenomenon, the long-term stability of the gel is good. Thermal stability experiments were conducted at 105 °C; the gelling solution was sealed in an ampoule bottle, and after gelation, it was taken out of the oven at the set time and the ampoule bottle containing the gel was opened. The mass of gel syneresis was measured using a ME403 electronic balance; due to the initial mass of the gel forming solution being 20 g, the syneresis rate of gel was equal to the mass of the separated water weighed by the balance divided by 20 g.

## Figures and Tables

**Table 1:** The main instruments.

Instrument Name	Instrument Type	Manufacturer
Precision digital display mixer	JJ-1	Jintan Jincheng Guosheng Experimental Instrument Factory (Changzhou, China)
Electronic balance	ME403	Mettler Toledo International Trade Co., Ltd. (Shanghai, China)
Projector display stand	GK-8000A	Ruiying Information Technology Co., Ltd. (Guangzhou, China)
Micro injection pump	100DX	Teledyne Isco, Inc. (Lincoln, Nebraska, USA)
Alcohol blowtorch	GW-6	Subei Experimental Instrument Co., Ltd. (Taizhou, China)
Constant temperature oven	DHG-9070A	Jinghong Experimental Equipment Co., Ltd. (Shanghai, China)

## Data Availability

Data are contained within the article.
